# Physical Origins of Memory Effects in a Non-Markovian Quantum Evolution

**DOI:** 10.3390/e27121207

**Published:** 2025-11-27

**Authors:** Shao-Cheng Hou, Yu-Han Zhou, Xing-Yuan Zhang, Xue-Xi Yi

**Affiliations:** 1 School of Science, Dalian Maritime University, Dalian 116026, China; 1120250952@dlmu.edu.cn (Y.-H.Z.); xyzhang@dlmu.edu.cn (X.-Y.Z.); 2 Center for Quantum Sciences and School of Physics, Northeast Normal University, Changchun 130024, China

**Keywords:** memory effects, system–environment correlations, non-Markovian quantum dynamics, non-Markovianity, Jaynes–Cummings model

## Abstract

We quantitatively investigate the physical origins of the non-Markovianity measure proposed in our previous work, which can be directly interpreted as memory effects, i.e., the dependence of a quantum system’s future evolution on its history. Using the properties of the trace norm and the trace distance, we find that the strength of memory effects in an evolution is upper (lower) bounded by the sum (difference) of two quantities. One originates from (bounded by) the change of environment state caused by the system, the other from (bounded by) the correlations between the system and the environment. The simulation results for the Jaynes–Cummings model show that the two origins may contribute to the memory effects in different manners, depending on the initial states of the environment and the system.

## 1. Introduction

Any real quantum system is open due to its interactions with the surrounding environment. Traditionally, the open quantum dynamics is often described by a Markovian process where the system’s evolution does not depend on its history, corresponding to a dynamical semigroup or a master equation in Lindblad form [1,2]. Microscopically, the environment in a Markov process is approximated to be uncorrelated with the system and unaffected by the system during an evolution [1,2] and thus be memoryless. However, in many scenarios, such as a strong system–environment coupling, a structured environment, or a small time scale, the memory effects play an important role and the Markovian description fails. The dynamics is then called non-Markovian. In recent decades, the study of non-Markovian quantum dynamics [3,4,5] has gained increasing attention. Specially, different measures [6,7,8,9,10] of quantum non-Markovainity have been proposed and applied to a variety of areas [11,12,13,14,15,16,17,18,19,20,21,22,23]. Besides the measure and applications of quantum non-Markovianity, the investigations of its origins have also attracted considerable interest [23,24,25,26,27,28,29,30,31,32,33,34,35], where the system–environment dynamics is considered. Particularly, the physical origins of non-Markovianity relevant to the Breuer–Laine–Pilo (BLP) measure [9] have been quantitatively studied [30,31,32,33,34,35] with the help of the properties of the trace distance as well as the trace norm. Two physical origins—the system–environment correlations and the change of the environment state—are found to be quantitatively connected with the BLP measure by several inequalities. Additionally, at least one of the origins is necessary for a nonzero non-Markovianity by the BLP measure.

Although significant works have been devoted to quantitatively understand the physical origins of quantum non-Markovianity, there are still questions to be addressed. For example, a nonzero non-Markovianity by the BLP measure is sufficient for the memory effects (meaning the dependence of the system’s future evolution on its history in this work) but not necessary [10]. Additionally, the existence of the system–environment correlations or (and) the change of the environment is not sufficient for a nonzero non-Markovianity by the BLP measure [33,34]. Moreover, the characterization of the BLP measure involves two evolutions (with two system initial states), making the interpretations of the relations between the non-Markovianity and its physical origins somewhat complicated. Therefore, closer quantitative connections between the memory effects and their physical origins in one evolution (with one system initial state) are worthy further exploration.

In Ref. [10], we propose a measure of quantum non-Markovianty whose physical meaning is interpreted as the dependence of the system’s future state (at $t_{2}$) on its history (from $t_{0}$ to $t_{1}$) in an evolution starting at $t_{0}$ ($t_{0}⩽t_{1}⩽t_{2}$). Recently, we used this interpretation to establish quantitative connections between the strength of memory effects and the characteristics in a superradiance [23] (dynamical decoupling [22]) process. The strength of the memory effects in Refs. [22,23] is characterized by the trace distance between the final state (at $t_{2}$) of an interested evolution and that of another evolution (with the same initial condition) where the environment state is reset at $t_{1}$. Motivated by the studies on the physical origins of the BLP measure [30,31,32,33,34,35], we use the properties of the trace norm and the trace distances to investigate the quantitative connections between the strength of memory effects (defined in Refs. [22,23]) and their physical origins in this work. We find that the strength of memory effects at $t_{2}$ is upper (lower) bounded by the sum (difference) of two quantities, which originate from the change of environment state at $t_{1}$ (caused by the system) and the system–environment correlations at $t_{1}$, respectively. Furthermore, the first (second) quantity is upper bounded by the change of the environment state (the system–environment correlations) at $t_{1}$. If one considers all the possible $t_{1}$ and $t_{2}$ in an evolution, the memory effects are expected to show up when at least one of the origins exists, unless the two quantities fully cancel out (or they are both zero) for any $t_{1}$ and $t_{2}$. The physical origins of memory effects in the limit $t_{2}\to t_{1}$ are also discussed. We illustrate our findings with the Jaynes–Cummings model where the two-level atom is regarded as the system and a single-mode field serves as an environment. With different environment (system) initial states, it is found that one of the origins may dominate the memory effects or two of the origins may collectively contribute to the memory effects. The first case enables us to test one of the physical origins through the memory effects. In the latter case, the two origins may contribute to the memory effects constructively or destructively, making the strength of the memory effects approach its upper or lower bound.

This paper is organized as follows: In Section 2, we review the non-Markovianity measure proposed in our previous work and its physical interpretation, which defines the strength of memory effects in an evolution. In Section 3, we quantitatively investigate the connections between the memory effects and its two physical origins. Our theory is illustrated in Section 4 with the Jaynes–Cummings model where different initial conditions are considered. Finally, we summarize our work in Section 5.

## 2. Non-Markovianity and Memory Effect

### 2.1. Measure of Non-Markovianity

The measure of non-Markovianity in Ref. [10] applies to a quantum process with a fixed (possibly time-dependent) system–environment Hamiltonian $H=H_{S}+H_{E}+H_{S\;E}$, a fixed environment initial state $\rho_{E_{I}}\left(t_{I}\right)$ and an arbitrary system initial state $\rho_{S}\left(t_{I}\right)$ where $t_{I}$ is an arbitrary initial time of an evolution. The possible evolutions start with an uncorrelated initial condition

(1) $$\begin{array}{c} \rho_{S\;E}\left(t_{I}\right)=\rho_{S}\left(t_{I}\right)⊗\rho_{E_{I}}\left(t_{I}\right). \end{array}$$

where $\rho_{E_{I}}\left(t_{I}\right)$ is governed by $H_{E}$ before an evolution and independent of the system. In general, it is given by [7]

(2) $$\begin{array}{c} \rho_{E_{I}}\left(t\right)=Te^{-i\int_{0}^{t}H_{E}\left(t^{\prime }\right)dt^{\prime }}\rho_{E_{I}}\left(0\right), \end{array}$$

where T is the time-ordering operator (ℏ=1 is used throughout the paper). The non-Markovianity measure is based on the dynamical map $T(t_{b},t_{a})$ that transfers $\rho_{S}\left(t_{a}\right)$ to $\rho_{S}\left(t_{b}\right)$ ($t_{a}⩽t_{b}$) with $t_{a}=t_{I}$, i.e.,

(3) $$\begin{array}{ccc} \rho_{S}\left(t_{b}\right) & = & T\left(t_{b},t_{a}\right)\rho_{S}\left(t_{a}\right)\\ & = & {\mathrm{Tr}}_{E}\left[U_{t_{b},t_{a}}\rho_{S}\left(t_{a}\right)⊗\rho_{E_{I}}\left(t_{a}\right)U_{t_{b},t_{a}}^{†}\right], \end{array}$$

where $U_{t_{b},t_{a}}=Te^{-i\int_{t_{a}}^{t_{b}}H\left(\tau \right)d\tau }$. By definition, the dynamical map $T(t_{b},t_{a})$ is always trace-preserving, completely positive (TPCP) and independent of the state it acts upon [2]. It is shown that the Markovian divisibility can be understood in terms of $T(t_{b},t_{a})$ [2,10] as

(4) $$\begin{array}{c} T\left(t_{2},t_{0}\right)=T\left(t_{2},t_{1}\right)T\left(t_{1},t_{0}\right), \end{array}$$

where $t_{0}⩽t_{1}⩽t_{2}$. With TPCP dynamical maps in Equation (4), its violation is manifested by the inequality

(5) $$\begin{array}{c} T\left(t_{2},t_{0}\right)\neq T\left(t_{2},t_{1}\right)T\left(t_{1},t_{0}\right), \end{array}$$

which is the criterion of non-Markovianity proposed in Ref. [10].

Consider a time interval $\left[t_{0},t_{max}\right]$ where interested evolutions happen and $t_{0}⩽t_{1}⩽t_{2}⩽t_{max}$: the measure of non-Markovianity $N_{M}$ is defined as the maximal difference between $T(t_{2},t_{0})$ and $T\left(t_{2},t_{1}\right)T\left(t_{1},t_{0}\right)$ while optimizing over $t_{1}$ and $t_{2}$ [10] ($t_{0}$ is fixed for simplicity). Specifically, it is calculated through the trace distance between the Choi–Jamiółkowski matrices [36,37] of $T(t_{2},t_{0})$ and $T\left(t_{2},t_{1}\right)T\left(t_{1},t_{0}\right)$, i.e.,

(6) $$\begin{array}{c} N_{M}=\max_{t_{1},t_{2}}D\left[\rho_{T(t_{2},t_{0})},\rho_{T\left(t_{2},t_{1}\right)T\left(t_{1},t_{0}\right)}\right]. \end{array}$$

Here, $D(\rho_{1},\rho_{2})$ represents the trace distance between two density matrices whose properties will be discussed in Section 3.1. The Choi–Jamiółkowski matrix of a dynamical map Λ is given by $\rho_{\Lambda }=\left(I⊗\Lambda \right)(|\psi_{A\;S}\rangle \langle \psi_{A\;S}|)$ where I is the identity map and $|\psi_{A\;S}\rangle =\frac{1}{\sqrt{d}}\sum_{i=1}^{d}{|i\rangle }_{A}{|i\rangle }_{S}$ is a maximally entangled state between an ancillary system *A* and the system *S* (both *d*-dimensional). Based on the dynamical maps $T(t_{b},t_{a})$, the measurement Equation (6) does not depend on the system initial states.

### 2.2. Interpretation as Memory Effects

The non-Markovianity criterion Equation (5) can be physically interpreted as memory effects [10,23], i.e., the dependence of the future (after $t_{1}$) state $\rho_{S}\left(t_{2}\right)$ on its history (from $t_{0}$ to $t_{1}$). To see this, let the left-hand and right-hand sides of Equation (5) act on a system initial state $\rho_{S}\left(t_{0}\right)$, which involves three evolutions:

(7) $$\begin{array}{ccc} A:\;\rho_{S}\left(t_{2}\right) & = & T\left(t_{2},t_{0}\right)\rho_{S}\left(t_{0}\right), \end{array}$$

(8) $$\begin{array}{ccc} B:\;\rho_{S}\left(t_{1}\right) & = & T\left(t_{1},t_{0}\right)\rho_{S}\left(t_{0}\right), \end{array}$$

(9) $$\begin{array}{ccc} C:\;\rho_{S}^{\prime }\left(t_{2}\right) & = & T\left(t_{2},t_{1}\right)\rho_{S}\left(t_{1}\right). \end{array}$$

If the inequality Equation (5) holds, there exists $\rho_{S}\left(t_{0}\right)$ such that $\rho_{S}\left(t_{2}\right)\neq \rho_{S}^{\prime }\left(t_{2}\right)$. Notice that in evolutions A and C, the system states at $t_{1}$ are both $\rho_{S}\left(t_{1}\right)$; however, their histories are different. That is, evolution A (starting at $t_{0}$) has a history from $t_{0}$ to $t_{1}$ encoded in $\rho_{S\;E}\left(t_{1}\right)=U_{t_{1},t_{0}}\rho_{S}\left(t_{0}\right)⊗\rho_{E_{I}}\left(t_{0}\right)U_{t_{1},t_{0}}^{†}$, while evolution C (starting at $t_{1}$) has no history before $t_{1}$. Therefore, the fact that $\rho_{S}\left(t_{2}\right)\neq \rho_{S}^{\prime }\left(t_{2}\right)$ demonstrates that the system’s history in $\left[t_{0},t_{1}\right]$ influences its future state at $t_{2}$ in evolution A. The memory effects can also be interpreted by focusing on the change of environment at $t_{1}$ in the process $\rho_{S}^{\prime }\left(t_{2}\right)=T\left(t_{2},t_{1}\right)T\left(t_{1},t_{0}\right)\rho_{S}\left(t_{0}\right)$. At the end of evolution B, the system–environment state is $\rho_{S\;E}\left(t_{1}\right)$, which is the same as in evolution A at $t_{1}$. After that, the environment is reset to $\rho_{E_{I}}\left(t_{1}\right)$ by $T(t_{2},t_{1})$ at the beginning of evolution C, i.e., $\rho_{S\;E}\left(t_{1}\right)\to \rho_{S}\left(t_{1}\right)⊗\rho_{E_{I}}\left(t_{1}\right)$. Thus, the system’s history information from $t_{0}$ to $t_{1}$ in evolution B is erased by $T(t_{2},t_{1})$. In contrast, the erasure of information never happens in evolution A. Therefore, $\rho_{S}\left(t_{2}\right)\neq \rho_{S}^{\prime }\left(t_{2}\right)$ demonstrates that the environment and the system–environment correlations remember the system’s history in $\left[t_{0},t_{1}\right]$, which influences the system’s future state at $t_{2}$ in evolution A.

Using the above interpretations, we quantify the strength of memory effects in an evolution with a particular system initial state $\rho_{S}\left(t_{0}\right)$ by

(10) $$\begin{array}{c} N_{M}\left[\rho_{S}\left(t_{0}\right)\right]=\max_{t_{1},t_{2}}D\left[\rho_{S}\left(t_{2}\right),\rho_{S}^{\prime }\left(t_{2}\right)\right] \end{array}$$

in Ref. [23]. The method is applied to a dissipative Tavis–Cummings model to study the influence of initial state on the superradiance characteristics [23]. Recently, we used its simpler form

(11) $$\begin{array}{c} N_{M}^{t_{1},t_{2}}\left[\rho_{S}\left(t_{0}\right)\right]=D\left[\rho_{S}\left(t_{2}\right),\rho_{S}^{\prime }\left(t_{2}\right)\right] \end{array}$$

to quantify the strength of memory effects with two specific time instants $t_{1}$ and $t_{2}$ in an evolution [22]. Equation (11) and its extended form are used to establish quantitative connections between the strength of memory effects and the characteristics in a dynamical decoupling process [22]. A nonzero $N_{M}^{t_{1},t_{2}}\left[\rho_{S}\left(t_{0}\right)\right]$ is a sufficient condition for the non-Markovianty criterion Equation (5) and of fundamental importance for understanding the physical origins of memory effects in an evolution. In the following, we use Equation (11) as a starting point to explore quantitative connections between the strength of memory effects and their origins.

## 3. Physical Origins of Memory Effects

### 3.1. Properties of the Trace Norm and the Trace Distance

Before quantitatively exploring the physical origins of memory effects, we review several properties of the trace norm and the trace distance. The trace distance of two density matrices $\rho_{1}$ and $\rho_{2}$ is defined as

(12) $$\begin{array}{c} D\left(\rho_{1},\rho_{2}\right)=\frac{1}{2}∥\rho_{1}-\rho_{2}∥, \end{array}$$

where $∥M∥=\mathrm{Tr}(\sqrt{M^{†}M})$ is the trace norm of an operator *M*. The trace norm satisfies the non-negativity ∥M∥⩾0 (equality achieved if M=0), the homogeneity

(13) $$\begin{array}{c} ∥\alpha M∥=|\alpha |∥M∥, \end{array}$$

where α is a real or complex constant, and the triangle inequality

(14) $$\begin{array}{c} ∥M+N∥⩽∥M∥+∥N∥ \end{array}$$

for two operators *M* and *N*. According to the above properties the trace norm, it can be seen that the following condition also holds:

(15) $$\begin{array}{c} |∥M∥-∥N∥|⩽∥M+N∥⩽∥M∥+∥N∥. \end{array}$$

As a metric, the trace distance is symmetric, i.e., $D\left(\rho_{1},\rho_{2}\right)=D\left(\rho_{2},\rho_{1}\right)$, non-negative,

(16) $$\begin{array}{c} 0⩽D(\rho_{1},\rho_{2})⩽1 \end{array}$$

with $D(\rho_{1},\rho_{2})=0$ iff $\rho_{1}=\rho_{2}$, and satisfies the triangle inequality

(17) $$\begin{array}{c} D\left(\rho_{1},\rho_{2}\right)⩽D\left(\rho_{1},\rho_{3}\right)+D\left(\rho_{2},\rho_{3}\right). \end{array}$$

Another important property is that the trace distance is contractive under trace-preserving operations E [38]:

(18) $$\begin{array}{c} D\left(E\rho_{1},E\rho_{2}\right)⩽D\left(\rho_{1},\rho_{2}\right). \end{array}$$

The operation E could be a partial trace operation, or a quantum channel, i.e., trace-preserving and completely positive dynamical map. Specially, the equality holds if the operation is unitary:

(19) $$\begin{array}{c} D\left(U\rho_{1}U^{†},U\rho_{2}U^{†}\right)=D\left(\rho_{1},\rho_{2}\right). \end{array}$$

Moreover, the trace distance is invariant with respect to the tensor product of another density matrix, i.e.,

(20) $$\begin{array}{c} D\left(\rho_{1}⊗\rho_{3},\rho_{2}⊗\rho_{3}\right)=D\left(\rho_{1},\rho_{2}\right). \end{array}$$

Physically, the trace distance can be interpreted as the the distinguishability of two quantum states.

### 3.2. Roles of Environment Change and System–Environment Correlations

As discussed above and mentioned in our previous works [10,23], in an evolution starting from the condition Equation (1) with $t_{I}=t_{0}$, the manifestation of memory effects $\rho_{S}\left(t_{2}\right)\neq \rho_{S}^{\prime }\left(t_{2}\right)$ originates from the difference between $\rho_{S\;E}\left(t_{1}\right)$ and $\rho_{S}\left(t_{1}\right)⊗\rho_{E_{I}}\left(t_{1}\right)$, which is related to the change of environment (caused by the system) and the existence of system–environment correlations. In the following, we quantitatively investigate the influences of the two origins on Equation (11) with the help of the properties of the trace norm and trace distance.

Considering the system–environment composite dynamics in Equation (11), $\rho_{S}\left(t_{2}\right)$ (the final state of evolution A) is given by

(21) $$\begin{array}{c} \rho_{S}\left(t_{2}\right)={\mathrm{Tr}}_{E}\left[U_{t_{2},t_{1}}\rho_{S\;E}\left(t_{1}\right)U_{t_{2},t_{1}}^{†}\right], \end{array}$$

where $\rho_{S\;E}\left(t_{1}\right)=U_{t_{1},t_{0}}\rho_{S}\left(t_{0}\right)⊗\rho_{E_{I}}\left(t_{0}\right)U_{t_{1},t_{0}}^{†}$. In contrast, $\rho_{S}^{\prime }\left(t_{2}\right)$ (the final state of evolution C) is

(22) $$\begin{array}{c} \rho_{S}^{\prime }\left(t_{2}\right)={\mathrm{Tr}}_{E}\left[U_{t_{2},t_{1}}\rho_{S}\left(t_{1}\right)⊗\rho_{E_{I}}\left(t_{1}\right)U_{t_{2},t_{1}}^{†}\right]. \end{array}$$

The composite state $\rho_{S\;E}\left(t_{1}\right)$ in Equation (21) can be expressed as two parts [2,24,32,33],

(23) $$\begin{array}{c} \rho_{S\;E}\left(t_{1}\right)=\rho_{S}\left(t_{1}\right)⊗\rho_{E}\left(t_{1}\right)+\chi \left(t_{1}\right), \end{array}$$

where $\rho_{S}\left(t_{1}\right)={\mathrm{Tr}}_{E}\left[\rho_{S\;E}\left(t_{1}\right)\right]$, $\rho_{E}\left(t_{1}\right)={\mathrm{Tr}}_{S}\left[\rho_{S\;E}\left(t_{1}\right)\right]$ and $\chi \left(t_{1}\right)=\rho_{S\;E}\left(t_{1}\right)-\rho_{S}\left(t_{1}\right)⊗\rho_{E}\left(t_{1}\right)$, which satisfies ${\mathrm{Tr}}_{E}\left[\chi \left(t_{1}\right)\right]=0$. The term $\chi (t_{1})$ represents all the classical and quantum correlations between the system and the environment at $t_{1}$ and satisfies

(24) $$\begin{array}{c} ∥\chi \left(t_{1}\right)∥=2D\left[\rho_{S\;E}\left(t_{1}\right),\rho_{S}\left(t_{1}\right)⊗\rho_{E}\left(t_{1}\right)\right] \end{array}$$

according to the definition of the trace distance. Inserting Equation (23) into Equation (21) and using the definition Equation (12), the strength of memory effects $N_{M}^{t_{1},t_{2}}\left[\rho_{S}\left(t_{0}\right)\right]$ satisfies

(25) $$\begin{array}{ccc} & & N_{M}^{t_{1},t_{2}}\left[\rho_{S}\left(t_{0}\right)\right]\\ & = & \frac{1}{2}∥\rho_{S}\left(t_{2}\right)-\rho_{S}^{\prime }\left(t_{2}\right)∥\\ & = & \frac{1}{2}∥{\mathrm{Tr}}_{E}\left\{U_{t_{2},t_{1}}\left[\rho_{S}\left(t_{1}\right)⊗\rho_{E}\left(t_{1}\right)+\chi \left(t_{1}\right)\right]U_{t_{2},t_{1}}^{†}\right\}-{\mathrm{Tr}}_{E}\left[U_{t_{2},t_{1}}\rho_{S}\left(t_{1}\right)⊗\rho_{E_{I}}\left(t_{1}\right)U_{t_{2},t_{1}}^{†}\right]∥\\ & = & ∥\frac{1}{2}{\mathrm{Tr}}_{E}\left\{U_{t_{2},t_{1}}\rho_{S}\left(t_{1}\right)⊗\left[\rho_{E}\left(t_{1}\right)-\rho_{E_{I}}\left(t_{1}\right)\right]U_{t_{2},t_{1}}^{†}\right\}+\frac{1}{2}{\mathrm{Tr}}_{E}\left[U_{t_{2},t_{1}}\chi \left(t_{1}\right)U_{t_{2},t_{1}}^{†}\right]∥ \end{array}$$

The first term in the last trace norm in Equation (25) is related to the change of the environment state at $t_{1}$ (caused by its interaction with the system), while the second term originates from the established system–environment correlations at $t_{1}$ due to the system–environment interaction. Equation (25) gives an analytic expression that captures how the system’s history before $t_{1}$ influences its future at $t_{2}$ through the two origins. It is seen that if the environment state at $t_{1}$ is not affected by the system [$\rho_{E}\left(t_{1}\right)=\rho_{E_{I}}\left(t_{1}\right)$] and not correlated with the systems [$\chi (t_{1})=0$], then the strength of memory effects after $t_{1}$ is always zero. Otherwise, $N_{M}^{t_{1},t_{2}}\left[\rho_{S}\left(t_{0}\right)\right]>0$ is expected unless the two terms are simultaneously zero or they fully cancel out for any $t_{2}$. If one considers all the possible $t_{1}$ and $t_{2}$ in an evolution, the memory effects are likely to show up if $\rho_{E}\left(t_{1}\right)\neq \rho_{E_{I}}\left(t_{1}\right)$ or $\chi (t_{1})\neq 0$, unless the two terms are simultaneously zero or they cancel out for any $t_{1}$ and $t_{2}$.

To explore the contributions of the two origins quantitatively, we refer to the quantity

(26) $$\begin{array}{c} E_{t_{2},t_{1}}=∥\frac{1}{2}{\mathrm{Tr}}_{E}\left\{U_{t_{2},t_{1}}\rho_{S}\left(t_{1}\right)⊗\left[\rho_{E}\left(t_{1}\right)-\rho_{E_{I}}\left(t_{1}\right)\right]U_{t_{2},t_{1}}^{†}\right\}∥ \end{array}$$

as the degree of influence of the environment change (at $t_{1}$) on the memory effects (at $t_{2}$) and the quantity

(27) $$\begin{array}{c} C_{t_{2},t_{1}}=∥\frac{1}{2}{\mathrm{Tr}}_{E}\left[U_{t_{2},t_{1}}\chi \left(t_{1}\right)U_{t_{2},t_{1}}^{†}\right]∥ \end{array}$$

as the degree of influence of the system–environment correlations (at $t_{1}$) on the memory effects (at $t_{2}$). Note that if one of them is zero, the other is equal to $N_{M}^{t_{1},t_{2}}\left[\rho_{S}\left(t_{0}\right)\right]$. Thus, alternatively, $E_{t_{2},t_{1}}$ or $C_{t_{2},t_{1}}$ can be understood as the strength of memory effects when only one origin in $\rho_{S\;E}\left(t_{1}\right)$ contributes to the memory effects. Applying the property Equation (15) to Equation (25), there is

(28) $$\begin{array}{c} |E_{t_{2},t_{1}}-C_{t_{2},t_{1}}|⩽N_{M}^{t_{1},t_{2}}\left[\rho_{S}\left(t_{0}\right)\right]⩽E_{t_{2},t_{1}}+C_{t_{2},t_{1}}. \end{array}$$

The condition demonstrates that, for an evolution starting with $\rho_{S}\left(t_{0}\right)$, the difference of $E_{t_{2},t_{1}}$ and $C_{t_{2},t_{1}}$ is sufficient for the emergence of memory effects and gives a lower bound of the strength of memory effects at $t_{2}$. Meanwhile, the sum of them provides an upper bound of the strength of memory effects at $t_{2}$.

The quantities $E_{t_{2},t_{1}}$ and $C_{t_{2},t_{1}}$ depend on $\rho_{S\;E}\left(t_{1}\right)$ and the time evolution operator $U_{t_{2},t_{1}}$. Using the properties of the trace distance, two weaker but simpler upper bounds for $E_{t_{2},t_{1}}$ and $C_{t_{2},t_{1}}$ can be obtained, which do not depend on $U_{t_{2},t_{1}}$. For $E_{t_{2},t_{1}}$, there is

(29) $$\begin{array}{ccc} E_{t_{2},t_{1}} & = & ∥\frac{1}{2}{\mathrm{Tr}}_{E}\left\{U_{t_{2},t_{1}}\rho_{S}\left(t_{1}\right)⊗\left[\rho_{E}\left(t_{1}\right)-\rho_{E_{I}}\left(t_{1}\right)\right]U_{t_{2},t_{1}}^{†}\right\}∥\\ & = & D\{{\mathrm{Tr}}_{E}\left[U_{t_{2},t_{1}}\rho_{S}\left(t_{1}\right)⊗\rho_{E}\left(t_{1}\right)U_{t_{2},t_{1}}^{†}\right],{\mathrm{Tr}}_{E}\left[U_{t_{2},t_{1}}\rho_{S}\left(t_{1}\right)⊗\rho_{E_{I}}\left(t_{1}\right)U_{t_{2},t_{1}}^{†}\right]\}\\ & ⩽ & D[U_{t_{2},t_{1}}\rho_{S}\left(t_{1}\right)⊗\rho_{E}\left(t_{1}\right)U_{t_{2},t_{1}}^{†},U_{t_{2},t_{1}}\rho_{S}\left(t_{1}\right)⊗\rho_{E_{I}}\left(t_{1}\right)U_{t_{2},t_{1}}^{†}]\\ & = & D[\rho_{S}\left(t_{1}\right)⊗\rho_{E}\left(t_{1}\right),\rho_{S}\left(t_{1}\right)⊗\rho_{E_{I}}\left(t_{1}\right)]\\ & ⩽ & D[\rho_{E}\left(t_{1}\right),\rho_{E_{I}}\left(t_{1}\right)]. \end{array}$$

The properties Equation (18) (E represents the partial trace here), Equations (19) and (20) are used in sequence in Equation (29) to obtain the upper bound $D[\rho_{E}\left(t_{1}\right),\rho_{E_{I}}\left(t_{1}\right)]$. This condition provides a quantitative connection between the change of the environment state (caused by the system) at $t_{1}$ and its influence on the strength of memory effects at $t_{2}$. Similarly, the upper bound of $C_{t_{2},t_{1}}$ is obtained by

(30) $$\begin{array}{ccc} C_{t_{2},t_{1}} & = & ∥\frac{1}{2}{\mathrm{Tr}}_{E}\left\{U_{t_{2},t_{1}}\chi \left(t_{1}\right)U_{t_{2},t_{1}}^{†}\right\}∥\\ & = & D\{{\mathrm{Tr}}_{E}\left[U_{t_{2},t_{1}}\rho_{S\;E}\left(t_{1}\right)U_{t_{2},t_{1}}^{†}\right],{\mathrm{Tr}}_{E}\left[U_{t_{2},t_{1}}\rho_{S}\left(t_{1}\right)⊗\rho_{E}\left(t_{1}\right)U_{t_{2},t_{1}}^{†}\right]\}\\ & ⩽ & D[U_{t_{2},t_{1}}\rho_{S\;E}\left(t_{1}\right)U_{t_{2},t_{1}}^{†},U_{t_{2},t_{1}}\rho_{S}\left(t_{1}\right)⊗\rho_{E}\left(t_{1}\right)U_{t_{2},t_{1}}^{†}]\\ & = & D[\rho_{S\;E}\left(t_{1}\right),\rho_{S}\left(t_{1}\right)⊗\rho_{E}\left(t_{1}\right)]. \end{array}$$

The properties Equation (18) (E represents the partial trace here) and Equation (19) are used in sequence in Equation (30). It is observed that the quantity $C_{t_{2},t_{1}}$ is upper bounded by the trace distance between $\rho_{S\;E}\left(t_{1}\right)$ and $\rho_{S}\left(t_{1}\right)⊗\rho_{E}\left(t_{1}\right)$, which can be regarded as a measure of correlations in $\rho_{S\;E}\left(t_{1}\right)$ [30]. Thus, Equation (30) connects the strength of correlations in $\rho_{S\;E}\left(t_{1}\right)$ to its influence on the strength of memory effects at $t_{2}$. The combination of Equations (29) and (30) leads to a weaker upper bound of $N_{M}^{t_{1},t_{2}}\left[\rho_{S}\left(t_{0}\right)\right]$:

(31) $$\begin{array}{c} N_{M}^{t_{1},t_{2}}\left[\rho_{S}\left(t_{0}\right)\right]⩽D\left[\rho_{E}\left(t_{1}\right),\rho_{E_{I}}\left(t_{1}\right)\right]+D\left[\rho_{S\;E}\left(t_{1}\right),\rho_{S}\left(t_{1}\right)⊗\rho_{E}\left(t_{1}\right)\right]. \end{array}$$

It reveals a clear and quantitative connection between the strength of memory effects and their two origins.

### 3.3. Comparison

In the following, we discuss the differences between our theory and that proposed in Ref. [33] by focusing on a quantum process where the evolutions of interest start with the condition Equation (1). In this case, the non-Markovianity of the quantum process is determined by the fixed environment initial state in Equation (1) and the system–environment Hamiltonian. Based on the theory in Ref. [33], a key quantitative relationship between the memory effects and their physical origins can be written as

(32) $$\begin{array}{c} B_{t_{2},t_{1}}-F_{t_{2},t_{1}}-D_{t_{1}}⩽\Delta D_{t_{2},t_{1}}⩽B_{t_{2},t_{1}}+F_{t_{2},t_{1}}-D_{t_{1}}. \end{array}$$

In the above condition, $\Delta D_{t_{2},t_{1}}=D\left[\rho_{S}^{1}\left(t_{2}\right),\rho_{S}^{2}\left(t_{2}\right)\right]-D\left[\rho_{S}^{1}\left(t_{1}\right),\rho_{S}^{2}\left(t_{1}\right)\right]$ is the time variation of the trace distance between the system states from two evolutions. The initial conditions of the two evolutions are $\rho_{S}^{1}\left(t_{0}\right)⊗\rho_{E_{I}}\left(t_{0}\right)$ and $\rho_{S}^{2}\left(t_{0}\right)⊗\rho_{E_{I}}\left(t_{0}\right)$. Note that $\Delta D_{t_{2},t_{1}}$ might be negative or positive, and $\Delta D_{t_{2},t_{1}}>0$ is the manifestation of memory effects by the BLP measure [9], interpreted as the information backflow from the environment to the system. The other quantities in Equation (32) are given by [33]

(33) $$\begin{array}{ccc} B_{t_{2},t_{1}} & = & ∥\frac{1}{2}{\mathrm{Tr}}_{E}\left\{U_{t_{2},t_{1}}\rho_{S}\left(t_{1}\right)⊗\left[\rho_{E}^{1}\left(t_{1}\right)-\rho_{E}^{2}\left(t_{1}\right)\right]U_{t_{2},t_{1}}^{†}\right\}\\ & & +\frac{1}{2}{\mathrm{Tr}}_{E}\left\{U_{t_{2},t_{1}}\left[\chi^{1}\left(t_{1}\right)-\chi^{2}\left(t_{1}\right)\right]U_{t_{2},t_{1}}^{†}\right\}∥, \end{array}$$

(34) $$\begin{array}{ccc} F_{t_{2},t_{1}} & = & \frac{1}{2}∥{\mathrm{Tr}}_{E}\left\{U_{t_{2},t_{1}}\left[\rho_{S}^{1}\left(t_{1}\right)⊗\rho_{E}^{1}\left(t_{1}\right)\right]U_{t_{2},t_{1}}^{†}\right\}-{\mathrm{Tr}}_{E}\left\{U_{t_{2},t_{1}}\left[\rho_{S}^{2}\left(t_{1}\right)⊗\rho_{E}^{1}\left(t_{1}\right)\right]U_{t_{2},t_{1}}^{†}\right\}∥,\;\; \end{array}$$

(35) $$\begin{array}{ccc} D_{t_{1}} & = & D[\rho_{S}^{1}\left(t_{1}\right),\rho_{S}^{2}\left(t_{1}\right)], \end{array}$$

where $\rho_{S}^{1,2}\left(t_{1}\right)={\mathrm{Tr}}_{E}\left[\rho_{S\;E}^{1,2}\left(t_{1}\right)\right]$, $\rho_{E}^{1,2}\left(t_{1}\right)={\mathrm{Tr}}_{S}\left[\rho_{S\;E}^{1,2}\left(t_{1}\right)\right]$, $\rho_{S\;E}^{1,2}\left(t_{1}\right)=U_{t_{1},t_{0}}\rho_{S}^{1,2}\left(t_{0}\right)⊗\rho_{E_{I}}\left(t_{0}\right)U_{t_{1},t_{0}}^{†}$ and $\chi^{1,2}\left(t_{1}\right)=\rho_{S\;E}^{1,2}\left(t_{1}\right)-\rho_{S}^{1,2}\left(t_{1}\right)⊗\rho_{E}^{1,2}\left(t_{1}\right)$. The quantity $B_{t_{2},t_{1}}$ represents the influence of $\rho_{E}^{1}\left(t_{1}\right)\neq \rho_{E}^{2}\left(t_{1}\right)$ plus $\chi^{1}\left(t_{1}\right)\neq \chi^{2}\left(t_{1}\right)$ on $D[\rho_{S}^{1}\left(t_{2}\right),\rho_{S}^{2}\left(t_{2}\right)]$ [33]. The quantity $F_{t_{2},t_{1}}$ represents the influence of $\rho_{S}^{1}\left(t_{1}\right)\neq \rho_{S}^{2}\left(t_{1}\right)$ on $D[\rho_{S}^{1}\left(t_{2}\right),\rho_{S}^{2}\left(t_{2}\right)]$ [33]. Furthermore, a weaker upper bound for $\Delta D_{t_{2},t_{1}}$ is given by [33]

(36) $$\begin{array}{c} \Delta D_{t_{2},t_{1}}⩽D\left[\rho_{S\;E}^{1}\left(t_{1}\right),\rho_{S}^{1}\left(t_{1}\right)⊗\rho_{E}^{1}\left(t_{1}\right)\right]+D\left[\rho_{S\;E}^{2}\left(t_{1}\right),\rho_{S}^{2}\left(t_{1}\right)⊗\rho_{E}^{2}\left(t_{1}\right)\right]+D\left[\rho_{E}^{1}\left(t_{1}\right),\rho_{E}^{2}\left(t_{1}\right)\right]. \end{array}$$

In Equations (32) and (36), $\Delta D_{t_{2},t_{1}}$ corresponds to $N_{M}^{t_{1},t_{2}}\left[\rho_{S}\left(t_{0}\right)\right]$ since both of them are connected with the strength of memory effects associated with $t_{1}$ and $t_{2}$ ($t_{0}$ is fixed). Accordingly, Equation (36) corresponds to Equation (31) since they both provide upper bounds of the strength of memory effects, which only depend on the system–environment states at $t_{1}$. Additionally, Equation (33) could be compared with the right-hand side of Equation (25) due to their formal and internal connections. For example, a nonzero $\rho_{E}^{1}\left(t_{1}\right)-\rho_{E}^{2}\left(t_{1}\right)$ in Equation (33) already implies that $\rho_{E}^{1}\left(t_{1}\right)\neq \rho_{E_{I}}\left(t_{1}\right)$ or $\rho_{E}^{2}\left(t_{1}\right)\neq \rho_{E_{I}}\left(t_{1}\right)$, i.e., the change of the environment state caused by the system in at least one evolution, which is also implied by Equation (25).

Within the above framework, the differences between our theory and that in Ref. [33] are summarized as follows. First, a sufficient condition for $\Delta D_{t_{2},t_{1}}>0$ (indicating memory effects) is $B_{t_{2},t_{1}}>F_{t_{2},t_{1}}+D_{t_{1}}$ according to Equation (32). That is, $B_{t_{2},t_{1}}$ must exceed a certain threshold determined by $\rho_{S}^{1}\left(t_{1}\right)-\rho_{S}^{2}\left(t_{1}\right)$ (implied in $F_{t_{2},t_{1}}$ and $D_{t_{1}}$) to display memory effects. In other words, $B_{t_{2},t_{1}}>0$ may not necessarily lead to memory effects under the theory in Ref. [33]. In contrast, a nonzero value of the right-hand side of Equation (25) is directly interpreted as memory effects in our work, thereby establishing a more direct connection between the memory effects and their origins. Second, two system initial states $\rho_{S}^{1}\left(t_{0}\right)$ and $\rho_{S}^{2}\left(t_{0}\right)$ are required in Equations (32) and (36). In contrast, one system initial state $\rho_{S}\left(t_{0}\right)$ is used in our theory. Besides offering formal simplicity, our theory enables us to investigate the physical origins of memory effects in a single evolution. Third, unlike Equation (32), the influences of the environment state change and the system–environment correlations on the memory effects are treated separately in our work, as seen in condition (28).

### 3.4. The Case $t_{2}\to t_{1}$

The above analysis focuses on the influence of the system’s history from $t_{0}$ to $t_{1}$ on its future state at $t_{2}$. Let $t_{2}=t_{1}+\Delta t$. It is seen that Δt⩾0 follows from the assumption $t_{0}⩽t_{1}⩽t_{2}$. In the limit Δt→0, there is $N_{M}^{t_{1},t_{2}}\left[\rho_{S}\left(t_{0}\right)\right]\to 0$ since $T\left(t_{2},t_{0}\right)\to T\left(t_{1},t_{0}\right)$ in Equation (7) and $T(t_{2},t_{1})\to I$ in Equation (). For an evolution where the system initial state is $\rho_{S}\left(t_{0}\right)$, to investigate the influence of the system’s history from $t_{0}$ to $t_{1}$ on its future state at the upcoming moment $t_{2}=t_{1}+\Delta t$ (Δt→0), we define $S^{t_{1}}\left[\rho_{S}\left(t_{0}\right)\right]$ as

(37) $$\begin{array}{c} S^{t_{1}}\left[\rho_{S}\left(t_{0}\right)\right]=\lim_{\Delta t\to 0}\frac{N_{M}^{t_{1},t_{2}}\left[\rho_{S}\left(t_{0}\right)\right]}{\Delta t}, \end{array}$$

which could be understood as a dynamical strength of memory effects that evolves with $t_{1}$. Since $N_{M}^{t_{1},t_{2}}\left[\rho_{S}\left(t_{0}\right)\right]⩾0$ and Δt⩾0, there is $0⩽S^{t_{1}}\left[\rho_{S}\left(t_{0}\right)\right]⩽\infty$ in general according to Equation (37). The quantitative influences of the environment change and the system–environment correlations on $S^{t_{1}}\left[\rho_{S}\left(t_{0}\right)\right]$ are further discussed as follows. Assuming that the system–environment Hamiltonian *H* is time dependent for simplicity, there is

(38) $$\begin{array}{ccc} & & S^{t_{1}}\left[\rho_{S}\left(t_{0}\right)\right]\\ & = & \lim_{\Delta t\to 0}\frac{1}{\Delta t}D\left[\rho_{S}\left(t_{1}+\Delta t\right),\rho_{S}^{\prime }\left(t_{1}+\Delta t\right)\right]\\ & = & \lim_{\Delta t\to 0}\frac{1}{2\Delta t}∥{\mathrm{Tr}}_{E}\left\{U_{\Delta t}\rho_{S}\left(t_{1}\right)⊗\left[\rho_{E}\left(t_{1}\right)-\rho_{E_{I}}\left(t_{1}\right)\right]U_{\Delta t}^{†}\right\}+{\mathrm{Tr}}_{E}\left[U_{\Delta t}\chi \left(t_{1}\right)U_{\Delta t}^{†}\right]∥ \end{array}$$

followed from Equation (25) where $U_{\Delta t}=e^{-iH\Delta t}$. Using the Baker–Campbell–Hausdorff formula

(39) $$\begin{array}{c} e^{\alpha A}Be^{-\alpha A}=B+\alpha \left[A,B\right]+\frac{\alpha^{2}}{2!}\left[A,\left[A,B\right]\right]+\ldots , \end{array}$$

with α=−iΔt, A=H, $B=\rho_{S}\left(t_{1}\right)⊗\left[\rho_{E}\left(t_{1}\right)-\rho_{E_{I}}\left(t_{1}\right)\right]$ or $\chi (t_{1})$, $S^{t_{1}}\left[\rho_{S}\left(t_{0}\right)\right]$ can be expressed as

(40) $$\begin{array}{ccc} \;\;\;\; & & \;\;\;\;S^{t_{1}}\left[\rho_{S}\left(t_{0}\right)\right]\\ \;\;\;\; & = & \;\;\;\;\lim_{\Delta t\to 0}\frac{1}{2\Delta t}∥{\mathrm{Tr}}_{E}\left\{\rho_{S}\left(t_{1}\right)⊗\left[\rho_{E}\left(t_{1}\right)-\rho_{E_{I}}\left(t_{1}\right)\right]\right\}+{\mathrm{Tr}}_{E}\left\{-i\Delta t\left[H,\rho_{S}\left(t_{1}\right)⊗\left[\rho_{E}\left(t_{1}\right)-\rho_{E_{I}}\left(t_{1}\right)\right]\right]\right\}\\ \;\;\;\; & & \;\;\;\;+{\mathrm{Tr}}_{E}\left[\chi \left(t_{1}\right)\right]+{\mathrm{Tr}}_{E}\left\{-i\Delta t\left[H,\chi \left(t_{1}\right)\right]\right\}∥, \end{array}$$

where we have kept terms with Δt and omitted those with higher orders of Δt in the limit Δt→0. In Equation (40), the first and third terms in the trace norm are both 0, and the coefficient −i in the second and fourth terms does not change the trace norm according to Equation (13). Therefore, Equation (40) can be further simplified as

(41) $$\begin{array}{c} S^{t_{1}}\left[\rho_{S}\left(t_{0}\right)\right]=∥\frac{1}{2}{\mathrm{Tr}}_{E}\left\{\left[H,\rho_{S}\left(t_{1}\right)⊗\left[\rho_{E}\left(t_{1}\right)-\rho_{E_{I}}\left(t_{1}\right)\right]\right]\right\}+\frac{1}{2}{\mathrm{Tr}}_{E}\left\{\left[H,\chi \left(t_{1}\right)\right]\right\}∥, \end{array}$$

where the first and second terms in the trace norm originate from the environment change and system–environment correlations, respectively. Let

(42) $$\begin{array}{ccc} E_{t_{1}}^{\prime } & = & ∥\frac{1}{2}{\mathrm{Tr}}_{E}\left\{\left[H,\rho_{S}\left(t_{1}\right)⊗\left[\rho_{E}\left(t_{1}\right)-\rho_{E_{I}}\left(t_{1}\right)\right]\right]\right\}∥, \end{array}$$

(43) $$\begin{array}{ccc} C_{t_{1}}^{\prime } & = & ∥\frac{1}{2}{\mathrm{Tr}}_{E}\left\{\left[H,\chi \left(t_{1}\right)\right]\right\}∥ \end{array}$$

be the degrees of influence of the two origins on $S^{t_{1}}\left[\rho_{S}\left(t_{0}\right)\right]$, respectively. Then, similar to Equation (28), the upper and lower bounds of $S^{t_{1}}\left[\rho_{S}\left(t_{0}\right)\right]$ are given by

(44) $$\begin{array}{c} |E_{t_{1}}^{\prime }-C_{t_{1}}^{\prime }|⩽S^{t_{1}}\left[\rho_{S}\left(t_{0}\right)\right]⩽E_{t_{1}}^{\prime }+C_{t_{1}}^{\prime } \end{array}$$

according to the property Equation (15). This condition shows that the difference of $E_{t_{1}}^{\prime }$ and $C_{t_{1}}^{\prime }$ is sufficient for the emergence of memory effects in the upcoming moment after $t_{1}$ and provides a lower bound of $S^{t_{1}}\left[\rho_{S}\left(t_{0}\right)\right]$. Meanwhile, $S^{t_{1}}\left[\rho_{S}\left(t_{0}\right)\right]$ is upper bounded by the sum of $E_{t_{1}}^{\prime }$ and $C_{t_{1}}^{\prime }$. Equation (44) is connected with condition Equation (29) by $E_{t_{1}}^{\prime }=\lim_{\Delta t\to 0}\frac{E_{t_{2},t_{1}}}{\Delta t}$, $C_{t_{1}}^{\prime }=\lim_{\Delta t\to 0}\frac{C_{t_{2},t_{1}}}{\Delta t}$ and its definition Equation (37).

## 4. Example

### 4.1. Model and Simulations

The aim of this section is to reveal how the environment change and system–environment correlations may influence the strength of memory effects in different manners. We consider the Jaynes–Cummings model where the two-level atom is regarded as the system and the single-mode field is regarded as its environment. The Hamiltonian is written as

(45) $$\begin{array}{ccc} H & = & H_{S}+H_{E}+H_{S\;E}\\ & = & \omega_{S}\sigma^{+}\sigma^{-}+\omega_{E}a^{†}a+g\left(\sigma^{+}a+\sigma^{-}a^{†}\right), \end{array}$$

where $\omega_{S}$ and $\omega_{E}$ are the transition frequencies of the atom and the single-mode field, respectively, and *g* is their coupling strength. $\sigma^{+}=\left|e\rangle \langle g\right|$ and $\sigma^{-}=\left|g\rangle \langle e\right|$ are the atomic raising and lowering operators where |e〉 (|g〉) represents the excited (ground) state of the atom. $a^{†}$ and *a* are the creation and annihilation operators of the single-mode field.

Despite its simple form, the dynamics of the Jaynes–Cummings model is rich, depending on the Hamiltonian *H*, the system–environment initial state and the evolution time. Accordingly, the environment change and system–environment correlations may influence the strength of memory effects in different manners, depending on the Hamiltonian *H*, the environment initial state $\rho_{E_{I}}\left(t_{0}\right)$, the system initial state $\rho_{S}\left(t_{0}\right)$ and the ranges of $t_{1}$ and $t_{2}$ ($t_{0}=0$ is used in this section). In this work, we consider the resonance case, i.e., $\omega_{S}=\omega_{E}$. In this condition, it is verified that our results, i.e., $N_{M}^{t_{1},t_{2}}\left[\rho_{S}\left(t_{0}\right)\right]$, $E_{t_{2},t_{1}}$, $C_{t_{2},t_{1}}$, $S^{t_{1}}\left[\rho_{S}\left(t_{0}\right)\right]$, $E_{t_{1}}^{\prime }$, $C_{t_{1}}^{\prime }$, etc., do not depend on the coupling strength $g/\omega_{S}$ ($t_{1}$ and $t_{2}$ in units of 1/g). The reason can be briefly explained as follows: In the resonance case, different coupling strengths $g/\omega_{S}$ lead to the same interaction picture Hamiltonian ${\tilde{H}}_{S\;E}=H_{S\;E}=g\left(\sigma^{+}a+\sigma^{-}a^{†}\right)$; meanwhile, our results are independent of whether the Schrödinger or interaction picture is chosen. In contrast, we find that the influences of the two origins on the memory effects highly depend on the environment initial state as well as the system initial state in our model.

In the following, we numerically calculate the system–environment time evolution operators $U_{t_{b},t_{a}}=e^{-iH(t_{b}-t_{a})}$, with which we further calculate the values of $N_{M}^{t_{1},t_{2}}\left[\rho_{S}\left(t_{0}\right)\right]$, $E_{t_{2},t_{1}}$ (and its upper bound) and $C_{t_{2},t_{1}}$ (and its upper bound) for different $t_{1}$ and $t_{2}$ and the values of $S^{t_{1}}\left[\rho_{S}\left(t_{0}\right)\right]$, $E_{t_{1}}^{\prime }$ and $C_{t_{1}}^{\prime }$ for different $t_{1}$. We will illustrate several typical results where the vacuum state |0〉, the coherent state

(46) $$\begin{array}{c} |\alpha \rangle =e^{-\frac{1}{2}{\left|\alpha \right|}^{2}}\sum_{n=0}^{\infty }\frac{\alpha^{n}}{\sqrt{n!}}|n\rangle \end{array}$$

(with a finite average photon number ${\left|\alpha \right|}^{2}$) and the thermal state

(47) $$\begin{array}{c} \rho^{\mathrm{th}}=\sum_{n=0}^{\infty }\frac{\bar{n}}{{\left(1+\bar{n}\right)}^{n+1}}\left|n\rangle \langle n\right| \end{array}$$

(with a finite average photon number $\bar{n}$) of the single-mode field are chosen as the environment initial states at $t_{0}$. |e〉 or $(|e\rangle +\left|g\rangle \right)/\sqrt{2}$ are used as the system initial states at $t_{0}$. Without the loss of generality, the Schrödinger picture Hamiltonian Equation (45) is used in our simulations with g=0.01$\omega_{S}$. For the case where the environment is initially in a coherent state at $t_{0}$, i.e., $\rho_{E_{I}}\left(t_{0}\right)=\left|\alpha \rangle \langle \alpha \right|$, the environment initial state at $t_{1}$ is obtained by $\rho_{E_{I}}\left(t_{1}\right)=e^{-i\omega_{E}a^{†}a\left(t_{1}-t_{0}\right)}\rho_{E_{I}}\left(t_{0}\right)$ according to Equation (2). For the vacuum state and thermal state, which are stationary under $H_{E}$, there is $\rho_{E_{I}}\left(t_{1}\right)=\rho_{E_{I}}\left(t_{0}\right)$. A truncated Fock space for the single-mode field is applied in our simulations. In each simulation, the maximum occupation number in the truncated space is large enough to guarantee the convergence of the results.

### 4.2. Results for $N_{M}^{t_{1},t_{2}}\left[\rho_{S}\left(t_{0}\right)\right]$

**Figure 1:**
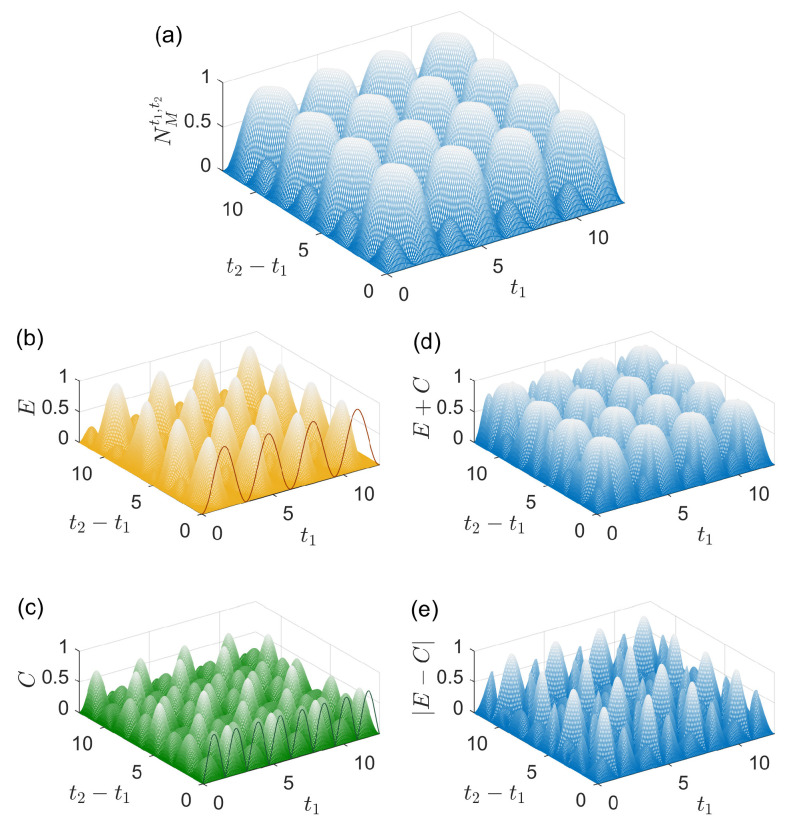
Physical origins of the memory effects in an evolution. (**a**) The strength of memory effects $N_{M}^{t_{1},t_{2}}\left[\rho_{S}\left(t_{0}\right)\right]$, (**b**) $E_{t_{2},t_{1}}$ plus its upper bound $D[\rho_{E}\left(t_{1}\right),\rho_{E_{I}}\left(t_{1}\right)]$ (solid line), (**c**) $C_{t_{2},t_{1}}$ plus its upper bound $D[\rho_{S\;E}\left(t_{1}\right),\rho_{S}\left(t_{1}\right)⊗\rho_{E}\left(t_{1}\right)]$ (solid line) and (**d**) $E_{t_{2},t_{1}}+C_{t_{2},t_{1}}$ and (**e**) $|E_{t_{2},t_{1}}-C_{t_{2},t_{1}}|$ as functions of $t_{1}$ and $t_{2}-t_{1}$ (both in units of 1/g). The environment initial state at $t_{0}$ is |0〉, and the system initial state at $t_{0}$ is |e〉.

In Figure 1, we illustrate the conditions Equations (26)–(30) where the system initial state is $\rho_{S}\left(t_{0}\right)=\left|e\rangle \langle e\right|$ and the environment initial state is $\rho_{E_{I}}\left(t_{0}\right)=\left|0\rangle \langle 0\right|$. $t_{1}$ and $t_{2}$ are in units of 1/g for all the figures in this paper. As shown in Figure 1a, the strength of memory effects $N_{M}^{t_{1},t_{2}}\left[\rho_{S}\left(t_{0}\right)\right]$ displays periodic oscillations as a function of either $t_{1}$ or $t_{2}-t_{1}$ with periods of π/g. The maximal value of $N_{M}^{t_{1},t_{2}}\left[\rho_{S}\left(t_{0}\right)\right]$ is 1, which demonstrates that the evolution is highly non-Markovian quantified by Equation (10). Additionally, it is observed that $N_{M}^{t_{1},t_{2}}\left[\rho_{S}\left(t_{0}\right)\right]=0$ when $t_{1},t_{2}-t_{1}=k\pi /g$ (k=0,1,2,…) due to the periodic evolutions of the reduced states of the system and the environment in the vacuum Rabi oscillations. The degree of influence of the environment change $E_{t_{2},t_{1}}$ (surface plot) and its upper bound $D[\rho_{E}\left(t_{1}\right),\rho_{E_{I}}\left(t_{1}\right)]$ (solid line) are shown in Figure 1b. The upper bound is zero at $t_{1}=k\pi /g$ (k=0,1,2,…) and attainable by $E_{t_{2},t_{1}}$ in Figure 1b. Similarly, the degree of influence of the system–environment correlations $C_{t_{2},t_{1}}$ (surface plot) and its upper bound $D[\rho_{S\;E}\left(t_{1}\right),\rho_{S}\left(t_{1}\right)⊗\rho_{E}\left(t_{1}\right)]$ (solid line) are shown in Figure 1c where the upper bound is zero at $t_{1}=k\pi /\left(2g\right)$ (k=0,1,2,…) and nearly attainable by $C_{t_{2},t_{1}}$. After carefully examining the results in Figure 1a–c, we find that, for the case $t_{1}=k\pi /\left(2g\right)$ (k=1,3,5,…), there exist $t_{1}$ and $t_{2}$ such that $N_{M}^{t_{1},t_{2}}\left[\rho_{S}\left(t_{0}\right)\right]=E_{t_{2},t_{1}}>0$ and $C_{t_{2},t_{1}}=0$, i.e., the memory effects may fully originate from the environment change for particular $t_{1}$ and $t_{2}$. Moreover, for the case $t_{2}-t_{1}=k\pi /g$ (k=0,1,2,…) where $N_{M}^{t_{1},t_{2}}\left[\rho_{S}\left(t_{0}\right)\right]=0$, there exist $t_{1}$ and $t_{2}$ such that $E_{t_{2},t_{1}}>0$ and $C_{t_{2},t_{1}}>0$, i.e., the influences of the two origins may fully cancel out for particular $t_{1}$ and $t_{2}$. In general, $N_{M}^{t_{1},t_{2}}\left[\rho_{S}\left(t_{0}\right)\right]$, $E_{t_{2},t_{1}}$ and $E_{t_{2},t_{1}}$ are simultaneously nonzero for an random pair of $t_{1}$ and $t_{2}$ as shown in Figure 1a–c, implying that the two origins collectively determine the strength of memory effects generally. The upper and lower bounds of $N_{M}^{t_{1},t_{2}}\left[\rho_{S}\left(t_{0}\right)\right]$, given by $E_{t_{2},t_{1}}+C_{t_{2},t_{1}}$ and $|E_{t_{2},t_{1}}-C_{t_{2},t_{1}}|$, are shown in Figure 1c and Figure 1d, respectively. It is observed that the dependence of $N_{M}^{t_{1},t_{2}}\left[\rho_{S}\left(t_{0}\right)\right]$ on $t_{1}$ and $t_{2}-t_{1}$ is different from those of its upper and lower bounds. However, there exist $t_{1}$ and $t_{2}$ that $N_{M}^{t_{1},t_{2}}\left[\rho_{S}\left(t_{0}\right)\right]\approx E_{t_{2},t_{1}}+C_{t_{2},t_{1}}$, e.g., when $N_{M}^{t_{1},t_{2}}\left[\rho_{S}\left(t_{0}\right)\right]$ is close to its maximum. In these cases, the influences of the two origins contribute constructively to the strength of memory effects.

**Figure 2:**
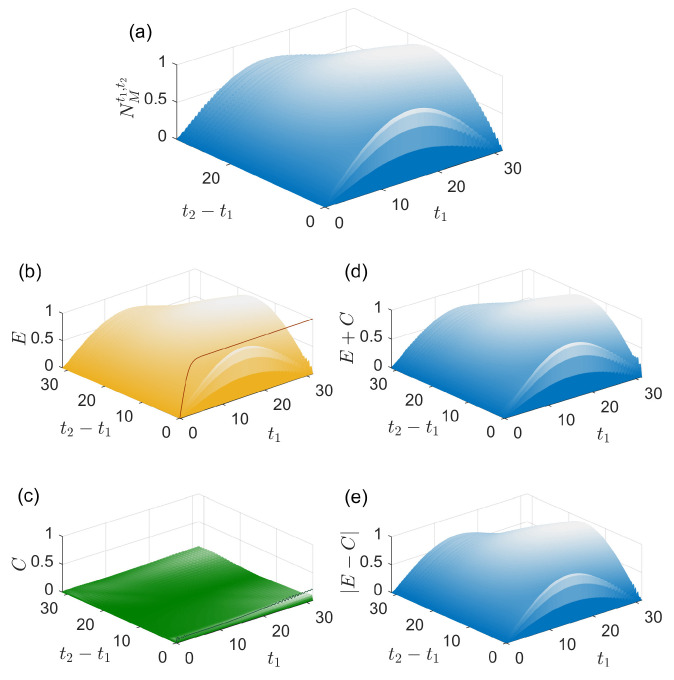
Physical origins of memory effects in an evolution. (**a**) The strength of memory effects $N_{M}^{t_{1},t_{2}}\left[\rho_{S}\left(t_{0}\right)\right]$, (**b**) $E_{t_{2},t_{1}}$ and plus upper bound $D[\rho_{E}\left(t_{1}\right),\rho_{E_{I}}\left(t_{1}\right)]$ (solid line), (**c**) $C_{t_{2},t_{1}}$ plus its upper bound $D[\rho_{S\;E}\left(t_{1}\right),\rho_{S}\left(t_{1}\right)⊗\rho_{E}\left(t_{1}\right)]$ (solid line) and (**d**) $E_{t_{2},t_{1}}+C_{t_{2},t_{1}}$ and (**e**) $|E_{t_{2},t_{1}}-C_{t_{2},t_{1}}|$ as functions of $t_{1}$ and $t_{2}-t_{1}$ (both in units of 1/g). The environment initial state at $t_{0}$ is a coherent state |α〉 with α=5 and the system initial state at $t_{0}$ is $(|e\rangle +\left|g\rangle \right)/\sqrt{2}$.

For the case where the environment at $t_{0}$ is in a coherent state |α〉 with α=5 and the system initial state is $(|e\rangle +\left|g\rangle \right)/\sqrt{2}$, the results are shown in Figure 2. As performed in Figure 1, $N_{M}^{t_{1},t_{2}}\left[\rho_{S}\left(t_{0}\right)\right]$, $E_{t_{2},t_{1}}$ with its upper bound, $C_{t_{2},t_{1}}$ with its upper bound, $E_{t_{2},t_{1}}+C_{t_{2},t_{1}}$ and $|E_{t_{2},t_{1}}-C_{t_{2},t_{1}}|$ are shown in Figure 2a–Figure 2e, respectively. The maximal strength of the memory effects in Figure 2a is $N_{M}^{t_{1},t_{2}}\left[\rho_{S}\left(t_{0}\right)\right]\approx 0.936$, which also exhibits strong memory effects in this evolution. In Figure 2b,c, $E_{t_{2},t_{1}}$ and $C_{t_{2},t_{1}}$ do not reach their upper bounds. In contrast to the results in Figure 1, the influence of the system–environment correlations on the memory effects is much weaker than that of the environment change for most pairs of $t_{1}$ and $t_{2}$ as shown in Figure 2b,c. Therefore, there is $N_{M}^{t_{1},t_{2}}\left[\rho_{S}\left(t_{0}\right)\right]\approx E_{t_{2},t_{1}}+C_{t_{2},t_{1}}\approx \left|E_{t_{2},t_{1}}-C_{t_{2},t_{1}}\right|\approx E_{t_{2},t_{1}}$ for the parameters in Figure 2, which means that the memory effects are dominated by the environment change (caused by the system) in Figure 2. Additionally, $N_{M}^{t_{1},t_{2}}\left[\rho_{S}\left(t_{0}\right)\right]$, $E_{t_{2},t_{1}}$ and $E_{t_{2},t_{1}}$ are all nonzero in Figure 2 except the trivial case $t_{1}=0,t_{2}=0$.

**Figure 3:**
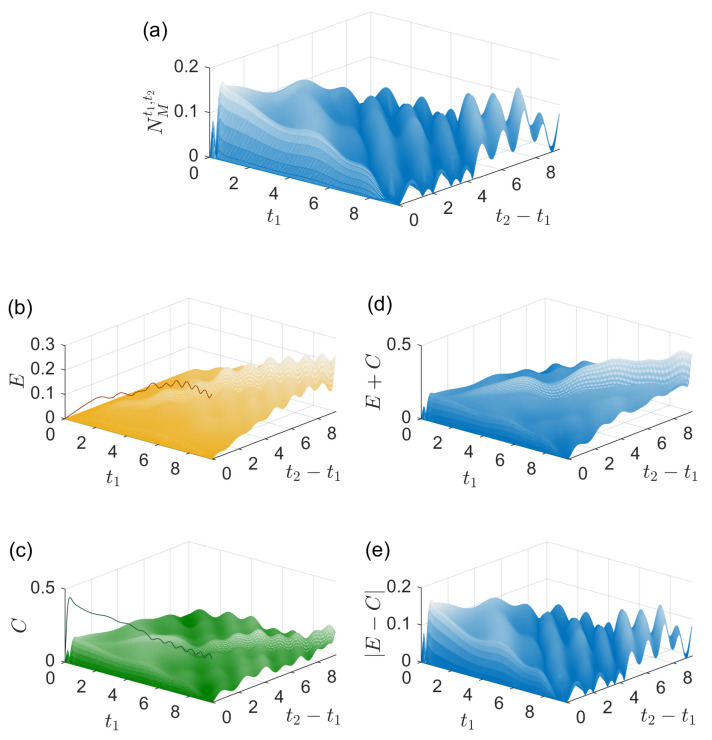
Physical origins of memory effects in an evolution. (**a**) The strength of memory effects $N_{M}^{t_{1},t_{2}}\left[\rho_{S}\left(t_{0}\right)\right]$, (**b**) $E_{t_{2},t_{1}}$ plus its upper bound $D[\rho_{E}\left(t_{1}\right),\rho_{E_{I}}\left(t_{1}\right)]$ (solid line), (**c**) $C_{t_{2},t_{1}}$ plus its upper bound $D[\rho_{S\;E}\left(t_{1}\right),\rho_{S}\left(t_{1}\right)⊗\rho_{E}\left(t_{1}\right)]$ (solid line) and (**d**) $E_{t_{2},t_{1}}+C_{t_{2},t_{1}}$ and (**e**) $|E_{t_{2},t_{1}}-C_{t_{2},t_{1}}|$ as functions of $t_{1}$ and $t_{2}-t_{1}$ (both in units of 1/g). The environment initial state at $t_{0}$ is a thermal state with $\bar{n}=20$ and the system initial state at $t_{0}$ is $(|e\rangle +\left|g\rangle \right)/\sqrt{2}$.

In the next case, we consider a thermal state $\rho^{\mathrm{th}}$ with an average photon number $\bar{n}=20$ as the environment initial state at $t_{0}$. The system initial state at $t_{0}$ is the same as that in Figure 2. The results are shown in Figure 3 where the meanings of all the quantities are the same as those in Figure 1. In Figure 3a, the maximal strength of memory effects is $N_{M}^{t_{1},t_{2}}\left[\rho_{S}\left(t_{0}\right)\right]\approx 0.168$, exhibiting weaker memory effects compared with the evolutions in Figure 1 and Figure 2. As seen in Figure 3b,c, the values of $E_{t_{2},t_{1}}$ and $C_{t_{2},t_{1}}$ do not reach their upper bounds. Moreover, the strengths of $E_{t_{2},t_{1}}$ and $C_{t_{2},t_{1}}$ are comparable in general. Interestingly, it is observed that the strength of memory effect $N_{M}^{t_{1},t_{2}}\left[\rho_{S}\left(t_{0}\right)\right]$ is close to its lower bound $|E_{t_{2},t_{1}}-C_{t_{2},t_{1}}|$ for any $t_{1}$ and $t_{2}$ in Figure 3a,d. That means the influences of the two origins contribute destructively to the strength of memory effects for the parameters in Figure 3. In addition, there exist $t_{1}$ and $t_{2}$ that $N_{M}^{t_{1},t_{2}}\left[\rho_{S}\left(t_{0}\right)\right]$ is close to zero, while $E_{t_{2},t_{1}}$ and $C_{t_{2},t_{1}}$ are both nonzero, e.g., the case when $t_{1}+\left(t_{2}-t_{1}\right)=t_{2}\approx 8.5/g$ as shown by the valley in Figure 3a, implying the influences of the two origins almost fully cancel out there.

### 4.3. Results for $S^{t_{1}}\left[\rho_{S}\left(t_{0}\right)\right]$

**Figure 4:**
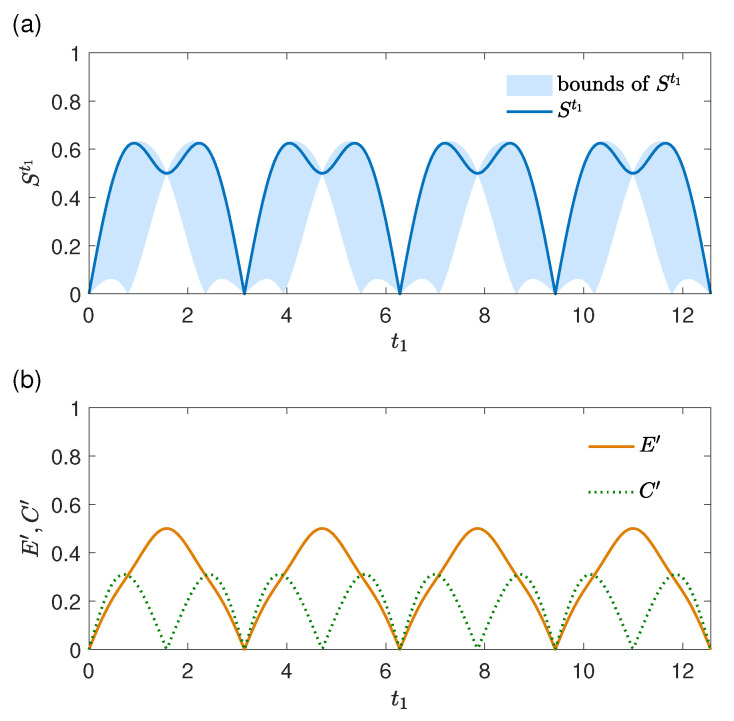
Physical origins of memory effects in an evolution in the limit $t_{2}\to t_{1}$. (**a**) The strength of memory effects $S^{t_{1}}\left[\rho_{S}\left(t_{0}\right)\right]$ (units of *g*) and its upper and lower bounds given by $E_{t_{1}}^{\prime }+C_{t_{1}}^{\prime }$ and $|E_{t_{1}}^{\prime }-C_{t_{1}}^{\prime }|$ and (**b**) $E_{t_{1}}^{\prime }$ and $C_{t_{1}}^{\prime }$ (both in units of *g*) as functions of $t_{1}$ (units of 1/g). The environment initial state at $t_{0}$ is |0〉, and the system initial state at $t_{0}$ is $(|e\rangle +\left|g\rangle \right)/\sqrt{2}$.

In this subsection, we illustrate the conditions Equations (41)–(44) with different system and environment initial states to show how the two origins may influence the memory effects in the limit $t_{2}\to t_{1}$ in different manners. The results for $\rho_{E}\left(t_{0}\right)=\left|0\rangle \langle 0\right|$ and $\rho_{S}\left(t_{0}\right)=\left|\phi \rangle \langle \phi \right|$ with $|\phi \rangle =(|e\rangle +\left|g\rangle \right)/\sqrt{2}$ are presented in Figure 4. The dynamical strength of memory effects $S^{t_{1}}\left[\rho_{S}\left(t_{0}\right)\right]$ (solid line, units of *g*), with $E_{t_{1}}^{\prime }+C_{t_{1}}^{\prime }$ and $|E_{t_{1}}^{\prime }-C_{t_{1}}^{\prime }|$ (the upper and lower edges of the shaded area, units of *g*), is plotted in Figure 4a as functions of $t_{1}$. It is observed that $S^{t_{1}}\left[\rho_{S}\left(t_{0}\right)\right]$ oscillates with period π/g, and its maximum is $S^{t_{1}}\left[\rho_{S}\left(t_{0}\right)\right]\approx 0.625$. The quantities $E_{t_{1}}^{\prime }$ and $C_{t_{1}}^{\prime }$ (units of *g*) are plotted in Figure 4b as functions of $t_{1}$, which oscillate with periods π/g and π/(2g), respectively. For $t_{1}=k\pi /\left(2g\right)$ (k=1,3,5,…), there is $C_{t_{1}}^{\prime }=0$ and $S^{t_{1}}\left[\rho_{S}\left(t_{0}\right)\right]\neq 0$; the memory effects at these instants are fully contributed by the environment change. In Figure 4a, $S^{t_{1}}\left[\rho_{S}\left(t_{0}\right)\right]$ is close to or near its upper bound $E_{t_{1}}^{\prime }+C_{t_{1}}^{\prime }$, implying the environment change and the correlations in $\rho_{S\;E}\left(t_{1}\right)$ contribute constructively to the dynamical strength of memory effects in general. Further simulation results show that if the initial condition is $\rho_{S\;E}\left(t_{0}\right)=\left|e\rangle \langle e\right|⊗\left|0\rangle \langle 0\right|$ as used in Figure 1, there are $E_{t_{1}}^{\prime }=0$ and $S^{t_{1}}\left[\rho_{S}\left(t_{0}\right)\right]=C_{t_{1}}^{\prime }$, i.e., the memory effects in the limit $t_{2}\to t_{1}$ are fully contributed by the system–environment correlations, even if the environment state changes as shown in Figure 1b. Meanwhile, $S^{t_{1}}\left[\rho_{S}\left(t_{0}\right)\right]$ oscillates with period π/(2g) and maximum 1. The above results demonstrate that the influences of the two origins on the memory effects may also highly depend on the system initial state.

**Figure 5:**
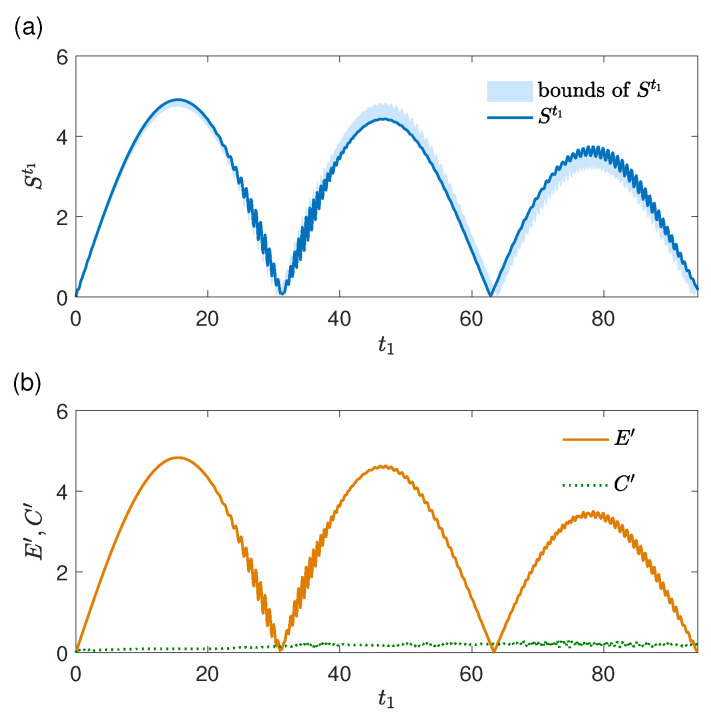
Physical origins of memory effects in an evolution in the limit $t_{2}\to t_{1}$. (**a**) The strength of memory effects $S^{t_{1}}\left[\rho_{S}\left(t_{0}\right)\right]$ (units of *g*) and its upper and lower bounds given by $E_{t_{1}}^{\prime }+C_{t_{1}}^{\prime }$ and $|E_{t_{1}}^{\prime }-C_{t_{1}}^{\prime }|$ and (**b**) $E_{t_{1}}^{\prime }$ and $C_{t_{1}}^{\prime }$ (both in units of *g*) as functions of $t_{1}$ (units of 1/g). The environment initial state at $t_{0}$ is a coherent state |α〉 with α=5, and the system initial state at $t_{0}$ is $(|e\rangle +\left|g\rangle \right)/\sqrt{2}$.

With the same system and environment initial states used in Figure 2, the results of $S^{t_{1}}\left[\rho_{S}\left(t_{0}\right)\right]$ and its upper and lower bounds, $E_{t_{1}}^{\prime }$ and $C_{t_{1}}^{\prime }$, are plotted in Figure 5. The maximal dynamical strength of the memory effects is $S^{t_{1}}\left[\rho_{S}\left(t_{0}\right)\right]\approx 4.91$, implying stronger memory effects compared with the evolution in Figure 4. Similar to the results in Figure 2, the influence of the system–environment correlations on $S^{t_{1}}\left[\rho_{S}\left(t_{0}\right)\right]$ is generally weaker compared with that of the environment change as shown in Figure 5b. Therefore, the dynamical strength of memory effects $S^{t_{1}}\left[\rho_{S}\left(t_{0}\right)\right]$ is dominated by the environment change (caused by the system). Accordingly, the upper and lower bounds are close, and $S^{t_{1}}\left[\rho_{S}\left(t_{0}\right)\right]\approx E_{t_{1}}^{\prime }$.

**Figure 6:**
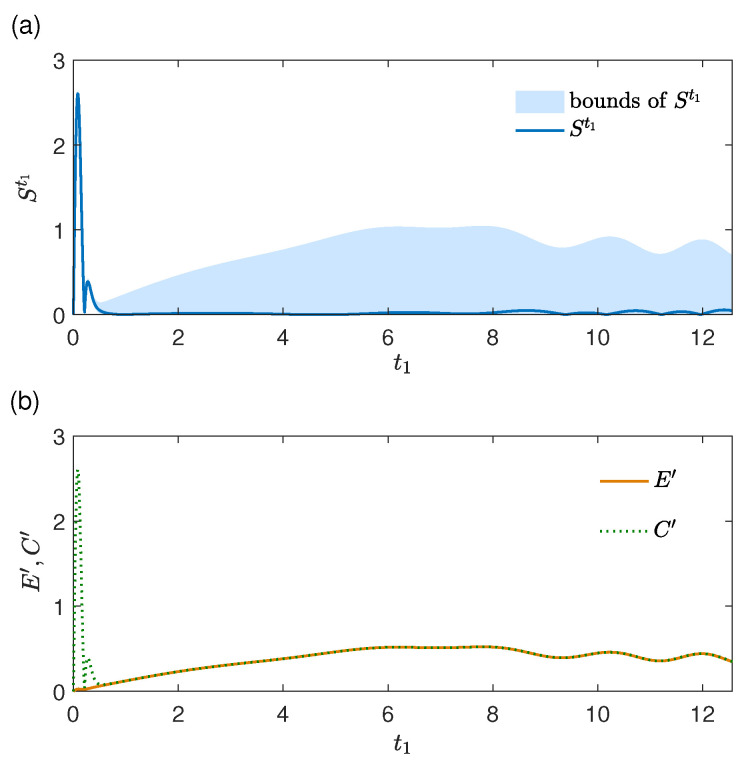
Physical origins of memory effects in an evolution in the limit $t_{2}\to t_{1}$. (**a**) The strength of memory effects $S^{t_{1}}\left[\rho_{S}\left(t_{0}\right)\right]$ (units of *g*) and its upper and lower bounds given by $E_{t_{1}}^{\prime }+C_{t_{1}}^{\prime }$ and $|E_{t_{1}}^{\prime }-C_{t_{1}}^{\prime }|$ and (**b**) $E_{t_{1}}^{\prime }$ and $C_{t_{1}}^{\prime }$ (both in units of *g*) as functions of $t_{1}$ (units of 1/g). The environment initial state at $t_{0}$ is a thermal with $\bar{n}=50$, and the system initial state at $t_{0}$ is $(|e\rangle +\left|g\rangle \right)/\sqrt{2}$.

In Figure 6, we consider the case where the environment state at $t_{0}$ is a thermal state with $\bar{n}=50$ and the system initial state at $t_{0}$ is $(|e\rangle +\left|g\rangle \right)/\sqrt{2}$. It is seen that the dynamical strength of memory effects $S^{t_{1}}\left[\rho_{S}\left(t_{0}\right)\right]$ and the influences of the two origins on $S^{t_{1}}\left[\rho_{S}\left(t_{0}\right)\right]$ depend on the range of $t_{1}$. In Figure 6a, $S^{t_{1}}\left[\rho_{S}\left(t_{0}\right)\right]$ reaches its maximum $S^{t_{1}}\left[\rho_{S}\left(t_{0}\right)\right]\approx 2.60$ when $t_{1}$ is small and then decreases significantly afterward. As seen in Figure 6b, in the early stage of the evolution, the influence of the environment change $E_{t_{1}}^{\prime }$ is weaker compared with $C_{t_{1}}^{\prime }$. So the dynamical strength of the memory effects is dominated by the system–environment correlations in this stage. Moreover, it is observed that $S^{t_{1}}\left[\rho_{S}\left(t_{0}\right)\right]$ is near its lower bound $|E_{t_{1}}^{\prime }-C_{t_{1}}^{\prime }|$ for all $t_{1}$ in Figure 6a, as $N_{M}^{t_{1},t_{2}}\left[\rho_{S}\left(t_{0}\right)\right]$ is in Figure 3. That means the two origins contribute destructively to the dynamical strength of memory effects $S^{t_{1}}\left[\rho_{S}\left(t_{0}\right)\right]$ in Figure 6. Specially, for $t_{1}≳0.6/g$, the values of $E_{t_{1}}^{\prime }$ and $C_{t_{1}}^{\prime }$ are almost the same, which leads to very weak memory effects as seen in Figure 6a.

## 5. Conclusions

We quantitatively investigate the connections between the memory effects in a non-Markovian evolution with a factorized system–environment initial state and their two physical origins: the first is the change of the environment state (caused by the system), and the second is the established system–environment correlations during the evolution. We identify two quantities $E_{t_{2},t_{1}}$ and $C_{t_{2},t_{1}}$, which can be understood as the strengths of the memory effect solely contributed by the first and the second origin, respectively. We find that the strength of memory effect $N_{M}^{t_{1},t_{2}}\left[\rho_{S}\left(t_{0}\right)\right]$ is upper (lower) bounded by the sum (difference) of $E_{t_{2},t_{1}}$ and $C_{t_{2},t_{1}}$. Furthermore, $E_{t_{2},t_{1}}$ and $C_{t_{2},t_{1}}$ are upper bounded by $D[\rho_{E}\left(t_{1}\right),\rho_{E_{I}}\left(t_{1}\right)]$ and $D[\rho_{S\;E}\left(t_{1}\right),\rho_{S}\left(t_{1}\right)⊗\rho_{E}\left(t_{1}\right)]$, respectively, representing the degree of the environment state change and the strength of system–environment correlations. The memory effects are expected to show up when there exists at least one of the origins, unless $E_{t_{2},t_{1}}$ and $C_{t_{2},t_{1}}$ cancel out (or they are simultaneous zero) for any $t_{1}$ and $t_{2}$. Thus, the strength of the memory effects is generally nonzero (maybe small) for an evolution without the Markovian approximation and reflects the validity of the Markovian approximation [10,23]. In the limit $t_{2}\to t_{1}$, the bound condition for $N_{M}^{t_{1},t_{2}}\left[\rho_{S}\left(t_{0}\right)\right]$ leads to an analogous bound condition, i.e., Equation (44), where $S^{t_{1}}\left[\rho_{S}\left(t_{0}\right)\right]=\lim_{\Delta t\to 0}\frac{N_{M}^{t_{1},t_{2}}\left[\rho_{S}\left(t_{0}\right)\right]}{\Delta t}$, $E_{t_{1}}^{\prime }=\lim_{\Delta t\to 0}\frac{E_{t_{2},t_{1}}}{\Delta t}$ and $C_{t_{1}}^{\prime }=\lim_{\Delta t\to 0}\frac{C_{t_{2},t_{1}}}{\Delta t}$.

We numerically calculated the bound conditions for the Jaynes–Cummings model with different environment and system initial states. The results confirm our findings and demonstrate that the two origins may influence the strength of the memory effects in different manners. For example, the memory effects may be contributed only (mainly) by one origin, or by the joint action of the two origins. In the first case, the strength of memory effects can be used to test the strength of one of the physical origin. In the second case, the two origins may contribute constructively or destructively to the strength of memory effects under certain conditions. Correspondingly, the strength of the memory effects may be close to its upper bound or lower bound. In our model, for particular $t_{1}$ and $t_{2}$, the influences of the two origins may fully cancel out, leading to zero memory effects. However, if we consider all the possible $t_{1}$ and $t_{2}$, the memory effects show up as we predicted. Our research provides deeper understanding of the physical mechanisms of quantum non-Markovianity, which is expected to advance the description, control and utilization of open quantum dynamics.

## Data Availability

The data presented in this study are available on request from the corresponding author due to privacy or ethical reasons.
